# Assessment of Associations Between Serum Lipoprotein (a) Levels and Atherosclerotic Vascular Diseases in Hungarian Patients With Familial Hypercholesterolemia Using Data Mining and Machine Learning

**DOI:** 10.3389/fgene.2022.849197

**Published:** 2022-02-09

**Authors:** Ákos Németh, Bálint Daróczy, Lilla Juhász, Péter Fülöp, Mariann Harangi, György Paragh

**Affiliations:** ^1^ Department of Internal Medicine, Faculty of Medicine, University of Debrecen, Debrecen, Hungary; ^2^ Doctoral School of Health Sciences, Faculty of Public Health, University of Debrecen, Debrecen, Hungary; ^3^ Institute for Computer Science and Control, Hungarian Academy of Sciences, (MTA SZTAKI), Budapest, Hungary; ^4^ Université Catholique de Louvain, INMA, Louvain-la-Neuve, Belgium

**Keywords:** lipoprotein(a), familial hypercholesterolemia, cardiovascular risk, data mining, atherosclerosis

## Abstract

**Background and aims:** Premature mortality due to atherosclerotic vascular disease is very high in Hungary in comparison with international prevalence rates, though the estimated prevalence of familial hypercholesterolemia (FH) is in line with the data of other European countries. Previous studies have shown that high lipoprotein(a)- Lp(a) levels are associated with an increased risk of atherosclerotic vascular diseases in patients with FH. We aimed to assess the associations of serum Lp(a) levels and such vascular diseases in FH using data mining methods and machine learning techniques in the Northern Great Plain region of Hungary.

**Methods:** Medical records of 590,500 patients were included in our study. Based on the data from previously diagnosed FH patients using the Dutch Lipid Clinic Network scores (≥7 was evaluated as probable or definite FH), we trained machine learning models to identify FH patients.

**Results:** We identified 459 patients with FH and 221 of them had data available on Lp(a). Patients with FH had significantly higher Lp(a) levels compared to non-FH subjects [236 (92.5; 698.5) vs. 167 (80.2; 431.5) mg/L, *p* < .01]. Also 35.3% of FH patients had Lp(a) levels >500 mg/L. Atherosclerotic complications were significantly more frequent in FH patients compared to patients without FH (46.6 vs. 13.9%). However, contrary to several other previous studies, we could not find significant associations between serum Lp(a) levels and atherosclerotic vascular diseases in the studied Hungarian FH patient group.

**Conclusion:** The extremely high burden of vascular disease is mainly explained by the unhealthy lifestyle of our patients (i.e., high prevalence of smoking, unhealthy diet and physical inactivity resulting in obesity and hypertension). The lack of associations between serum Lp(a) levels and atherosclerotic vascular diseases in Hungarian FH patients may be due to the high prevalence of these risk factors, that mask the deleterious effect of Lp(a).

## Introduction

In familial hypercholesterolemia, significantly elevated low-density lipoprotein-cholesterol (LDL-C) levels increase cardiovascular risk. A previous study in Norway showed that the life expectancy of individuals with heterozygous familial hypercholesterolemia (FH) was 15 years shorter than that of the average Norwegian population (Mundal et al., 2014). Different study pointed out that 25% of women and 50% of men with heterozygous FH had cardiovascular complications (McCrindle and Gidding, 2016). Compared to healthy individuals the risk of coronary artery disease is 3.5–16 times higher in patients with FH (Hovingh and Kastelein, 2016), while the risk of peripheral vascular disease was found to be elevated by 5–10 times in these individuals (Kroon et al., 1995; Hutter et al., 2004). In 1963, during his research on blood group antigens, Köre Berg discovered a new lipoprotein system, later named lipoprotein(a) (Lp(a)) (BERG, 1963). Lp(a) is an LDL-like lipoprotein particle produced by the liver. Its major lipoprotein is apolipoprotein B100 (apoB100), to which an apo (a) glycoprotein is covalently bound (Marcovina and Koschinsky, 1998; Anuurad et al., 2006). Association with proteoglycan and fibronectin molecules on the surface of endothelial cells Lp(a) can enter the subendothelial space (Stulnig et al., 2019). Phospholipids on the surface of Lp(a) can be oxidized by reactive free radicals produced by lipoxygenases, myeloperoxidases, and nicotinamide adenine dinucleotide phosphate oxidase (Ferretti et al., 2018). Oxidized phospholipids bound to Lp(a) enhance the production and expression of inflammatory cytokines and chemokines in vascular wall cells promoting the accumulation of monocytes from the circulation in the subendothelial space. Oxidized phospholipids trigger the endocytosis of Lp(a) through the binding to scavenger receptors of macrophages, as well as the migration of vascular smooth muscle cells from the media to the intima, ultimately leading to endothelial dysfunction (Wu et al., 2004; Tsimikas and Witztum, 2008). In addition, Lp(a) competitively inhibits plasminogen plasmin conversion and its binding to fibrin, and thus thrombolysis. Apolipoprotein (a) decreases plasminogen activator-1 levels by increasing the expression of the plasminogen activator-1 inhibitor. Thus, Lp(a) elicits proatherogenic, proinflammatory and prothrombotic effects (van der Valk et al., 2016). Analysis of 31 prospective studies showed a 1.5-fold increase of relative cardiovascular risk in individuals with Lp(a) levels in the upper third compared with those in the lower third (Bennet et al., 2008). A meta-analysis of 36 prospective studies involving 126,634 individuals showed that Lp(a) concentration was associated with the risk of cardiovascular disease as well as stroke (Erqou et al., 2009). Nordestgaard et al. recommended that Lp(a) should be measured in patients with moderate to high cardiovascular risk. (Nordestgaard et al., 2010). After LDL reduction, a reduction in Lp(a) serum levels below 50 mg/dl (500 mg/L) is recommended as a secondary priority. Previous studies have suggested that high levels of Lp(a) are more common in individuals with FH, further increasing the cardiovascular risk in these patients (Clarke et al., 2009; Kamstrup et al., 2009). FH is still underestimated and underdiagnosed in the regions of Central, Eastern and Southern Europe. However, during the last few years, the international ScreenPro Project achieved significant improvement of screening, diagnosis, and treatment of FH in these countries (Ceska et al., 2019). Based on medical and statistical records of two major hospitals in the Northern Great Plain region of Hungary, recently we identified patients with a possible diagnosis of FH using data mining methods. Investigating medical records of 1,342,124 patients the estimated prevalence of FH was found to be 1:340, which is in line with the prevalence data of some other European countries (Paragh et al., 2018).

In the present study, we examined the prevalence of high serum Lp(a) levels and their potential impact on atherosclerotic vascular complications in individuals with FH. We hypothesized that the prevalence of increased serum Lp(a) will be higher in FH patients, which can be associated with higher risk of atherosclerotic vascular complications including cardiovascular (CAD), cerebrovascular (CeVD) and peripheral arterial diseases (PAD), aortic valve stenosis (AoS), and might also be associated with the risk of deep vein thrombosis (DVT).

## Patients and Methods

### Screening Patients for FH Diagnosis

Described in our previous paper (Paragh et al., 2018) data mining methods are, an ideal way to screen for FH in mass hospital data, though the range for potential FH patients was still wide. To narrow our finding, we used cutting edge machine learning techniques. First, we decided to use the Dutch Lipid Network Criteria System (DLNCS) to teach how to recognize FH patients in order to find the most homogeneous patient group. We used the scores of the DLNCS for patient input and four machine learning model groups (feedforward multi-layer perceptron with ReLU (Rectified Linear Unit) activations (Montufar et al., 2014), gradient boosting (Friedman, 2001; Chen and Guestrin, 2016), support vector machines with RBF (radial basis function) kernel (Tan et al., 2016) and binary linear regression (Tan et al., 2016) for training. The training feature space included patient blood test results (with 70 most common test types), diagnostic data (ICD-10 3-digit diagnosis) and textual history data. Boosted trees worked the best similarly to other nonstructural datasets as in (Kerepesi et al., 2018). Then we majorly improved our textual analysis (to collect patient history and family history data more thoroughly and also to get detailed statistics on secondary medical conditions like hypertension or smoking for proper analysis) with Natural Language Processing (NLP) (see details in next subsection).

The best overall training results we achieved with DLNCS scores were 7+ and 8+, so we decided we consider everyone as an FH patient who possessed 7 or more within the score system, which is also entirely in line with the key concept of DLNCS.

Clinical characteristics and laboratory data of the study population are summarized in Table 1.

**TABLE 1:** Clinical and laboratory data of the study populations. Data are presented as median (lower-upper quartile).

	nonFH	FH	*p*
Number of patients	590,041	459	
Age (years)	40.0 (22.2–59.0)	53.2 (39.3–59.7)	<0.05
Male/Female (%)	42.8/57.2	39.2/60.8	n.s.
Cholesterol (mmol/L)	4.94 (4.21–5.73)	7.03 (6.10–8.40)	<0.001
Triglyceride (mmol/L)	1.35 (0.96–1.96)	1.96 (1.41–3.03)	<0.01
LDL-C (mmol/L)	2.95 (2.34–3.62)	4.80 (3.91–5.90)	<0.001
HDL-C (mmol/L)	1.32 (1.09–1.62)	1.41 (1.16–1.72)	<0.05
Urea (mmol/L)	5.10 (4.02–6.50)	5.43 (4.40,6.63)	n.s.
Creatinine (µmol/L)	68.3 (57.1–83.2)	69.1 (58.5–82.2)	n.s.
eGFR (mL/min/1.73m2)	90.0 (78.1–90.0)	88.8 (80.4–90.0)	n.s.
AST (GOT) (U/L)	21.0 (17.5–28.1)	21.8 (18.3–27.5)	n.s.
ALP (GPT) (U/L)	19.6 (14.0–29.2)	24.0 (16.5–33.0)	n.s.
GGT (U/L)	26.1 (16.1–48.2)	32.7 (20.2–58.9)	<0.05
Uric acid (µmol/L)	292 (235–357)	324 (266–384)	<0.05
Lipoprotein(a) (mg/L)	167 (80.2; 431.5)	236 (92.5; 698.5)	<0.01

### Identifying Cardiovascular Risk Factors and Data on Laboratory Parameters

Since our data set contains several types of data structures, we first created a common representation as a preparation for statistical analysis. The main reason for such a structure is to detect “properties” in any form. For example, high blood pressure could appear in the textual data in several forms as expressions, as a parameter or derived from actual measurements. We developed tools to extract the designated data from the available multiple sources. An additional challenge is data cleansing, especially filling missing or corrupted data parts and treating the various types of corrupted data differently. For example, in case of binary variables filling gaps with the mean value is misleading and should be avoided in any case. These special cases were handled mainly by regular expressions. Given the size of the data, we developed additional serializing and streaming methods to optimize the flexible final query engine which can handle incomplete data and may detect “properties” based on deduction. For textual information included preprocessing steps in the following order: parsing, stemming, stop word filtering, dictionary building with unigrams and bigrams after a by hand cleaning of expressions or terms. The cleaning contained a ranking of terms based on TF-IDF (term frequency-inverse document frequency) and word embeddings. As word embeddings available in Hungarian language are trained on traditional corpora, we needed to build our own language model based on the textual data. For this we have built a Gated Recurrent Unit model (Chung et al., 2014), a special recurrent neural network, based on the cleaned unigrams and bigrams as dictionaries. The final data structure contained the following appearances: a “property” was assigned to a patient if one of the following events happened: either it was detected by a regular expression, or it has high probability given the language model as an expression or based on the laboratory measurements it was directly true.

### Lp(a) Measurement

Lp(a) levels were determined from fresh sera with a Cobas c501 analyzer (Roche Ltd., Mannheim, Germany) according to the manufacturer’s instructions at the University of Debrecen Department of Laboratory Medicine. The reference range of Lp(a) is <300 mg/L.

### Structuring the Study Population

**FIGURE 1:**
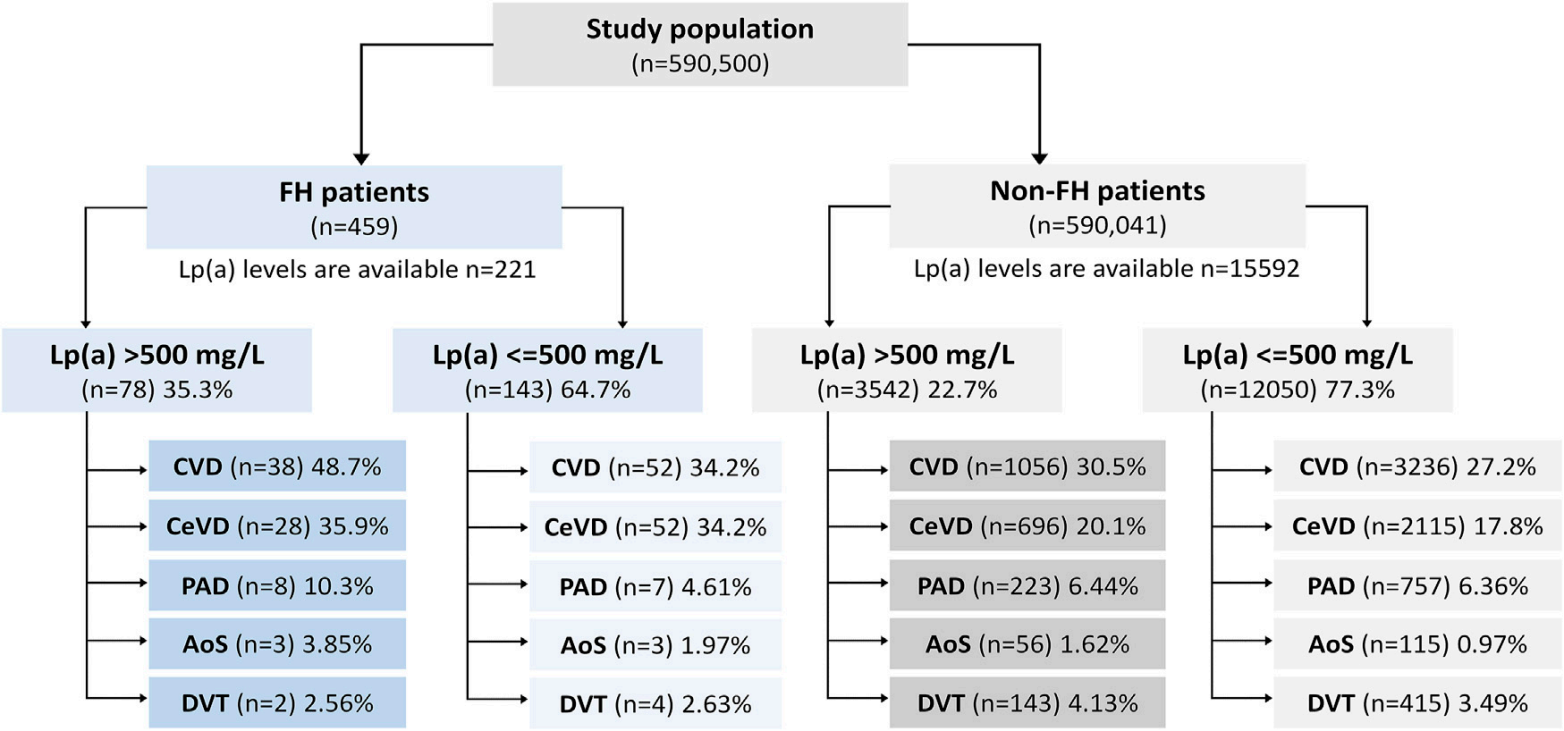
Flowchart showing the structure of the study population, number of enrolled patients, and how the patients were divided into the groups, and subgroups.

Figure 1 demonstrates a flowchart showing the structure of the study population, number of enrolled patients, and how the patients were divided into the groups, and subgroups.

### Statistical Analysis

We used anonymous patient record data from the hospital information system run by University of Debrecen Clinical Center’s hospital information system. The data was originally available in an HL7 format and has been already partially cleaned and preprocessed for data mining and machine learning purposes by a contractual cooperating partner of the university (Aesculab Medical Solutions, Black Horse Group Ltd.). We leveraged this database as starting point, so we did not have to deal with the system errors of original hospital data recording). The data included 8 complete years (from 2007 to 2014) and the entire patient record database of the clinical center with all textual, diagnostic and laboratory details. The data was extracted via queries from the PostgreSQL 13.x database and resulted in huge textual files which were the kick off for further statistical analysis. The studied population covered all patients treated in the University of Debrecen during these 8 years resulting in a number of 590,500 patients of which 288,591 had clinical laboratory tests available with an average of 34.03 and a median of 33.0 tests per person. The study data included all departments and all inpatient and outpatient data available during the aforementioned period.

Statistical analysis has been carried out with Python supported data mining packages. Data cleaning and preprocessing were done using Python 3.8, IPython 7.29, Cython 0.29, Pandas 0.23 and Numpy 1.22 under Conda 4.10 environment with Dask. Machine learning for refining data selection and deep textual analysis leveraged SciKit-Learn 1.0 and Pytorch 1.09. In general, for the base for statistical analysis we created a “healthy patient” pool, patients who do not suffer from any conditions (high LDL, low HDL, hypertension, diabetes, obesity, smoking, not following statin treatment) that might be associated with atherosclerotic vascular disease. Statistical significance analysis was performed with unpaired t-tests keeping 95% significance level. Statistical figures have been created with MatPlotLib 3.5 software package.

## Results

**FIGURE 2:**
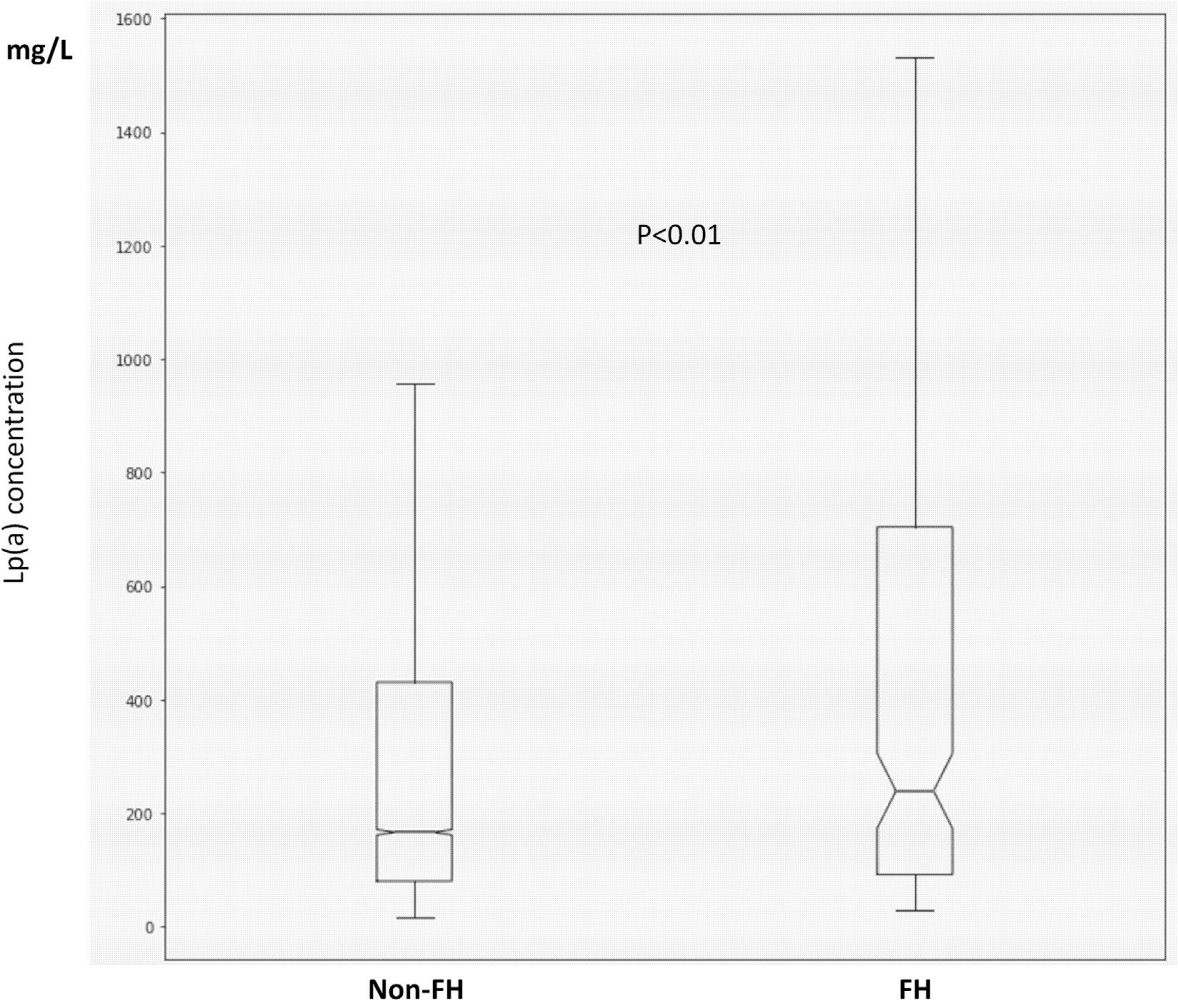
Boxplots and whiskers of serum lipoprotein(a) levels in FH and non-FH patients. The length of the box represents the interquartile range (IQR), the horizontal line in the box interior represents the median, the whiskers represent the 1.5 IQR of the 25th quartile or 1.5 IQR of the 75th quartile.

We identified 459 patients with FH, out of which 221 had data available on serum Lp(a) levels. Patients with FH had significantly higher Lp(a) levels compared to non-FH subjects [236 (92.5; 698.5) vs. 167 (80.2; 431.5) mg/L, *p* < .01] (Figure 2). Significantly higher Lp(a) levels were found in females compared to males with FH [266 (108–875) vs. 182 (73.1–648) mg/L, *p* < .05]. Similar differences were observed in those without FH [179 (81.5–458) vs. 152 (80.7–386) mg/L, *p* < .01]. 35.3% of FH patients had Lp(a) levels exceeding >500 mg/L. Atherosclerotic complications were significantly more frequent in FH patients compared to those without FH (46.6 vs. 13.9%). However, contrary to several other previous studies, we could not find significant associations between serum Lp(a) levels and atherosclerotic vascular diseases in the studied Hungarian FH patient group. Therefore, we determined the prevalence of other cardiovascular risk factors in FH and in non-FH patients. We found the prevalence of hypertension, smoking, obesity, and hyperuricemia extremely elevated in the FH group. Furthermore, the prevalence of diabetes and low HDL-C level were also increased compared to the non-FH population (Table 2).

**TABLE 2:** Prevalence of cardiovascular risk factors in the whole study population, in various atherosclerotic vascular diseases and in deep vein thrombosis.

	Non-FH	FH	CVD	CeVD	PAD	AoS	DVT
high Lp(a) (%)	23.0	35.3	25.7	25.8	23.8	33.9	26.2
hypertension (%)	24.5	86.3	77.8	82.6	61.1	85.0	64.2
diabetes (%)	5.16	17.4	19.3	24.4	14.7	23.0	15.7
smoking (%)	9.25	66.4	28.0	32.2	30.6	28.7	25.0
obesity (%)	10.2	42.0	31.4	36.1	26.3	33.5	38.2
hyperuricemia (%)	5.78	41.2	19.2	24.4	21.0	22.1	24.7
low HDL-C (%)	2.12	11.8	8.59	9.23	6.17	9.79	7.96
male gender (%)	42.7	39.2	48.1	46.5	26.6	57.8	42.4
age >60 ys (%)	23.0	20.0	54.8	64.7	33.7	77.8	50.5
no statin therapy (%)	88.3	12.9	46.6	45.3	56.2	35.6	68.8

Evaluating this population, the most common risk factors in patients with atherosclerotic vascular disease were hypertension, male gender, age >60 years and the lack of statin treatment. High prevalence of hypertension, male gender, age >60 years and the lack of statin treatment were found in patients with CeVD and PAD. In patients with AoS, the prevalence of hypertension and age >60 were extremely increased. Interestingly, similar risk factor pattern was detected in patients with DVT. The highest prevalence of elevated Lp(a) level was found in patients with AoS (Table 2).

We also detected the prevalence of the above-mentioned risk factors in FH patients with low and high Lp(a) levels. Although the prevalence of obesity was increased, and the prevalence of low HDL-C level was decreased in FH patients with high Lp(a) levels, there were no significant differences between the two groups. It must be noted that the ratio of individuals on statin treatment was markedly higher in FH patients with low Lp(a) level. Calculating the prevalence of risk factors, we found that the prevalence of high Lp(a) levels was increased in females, while the prevalence of smoking and hypertension were decreased in males in the non-FH population. In FH patients, the prevalence of high Lp(a) was tended to be augmented in females; however, this difference did not reach statistical significance similarly to the other risk factors (Table 3).

**TABLE 3:** Prevalence of cardiovascular risk factors in the whole study population, in FH patients and in FH patients with low and high (>500 mg/dl) Lp(a) levels.

	Non-FH	FH	FH with low Lp(a)	FH with high Lp(a)
Whole study population				
high Lp(a) (%)	23.0	35.3	—	—
hypertension (%)	24.5	86.3	86.8	87.2
diabetes (%)	5.16	17.4	16.4	12.8
smoking (%)	9.25	66.4	69.7	76.9
obesity (%)	10.2	42.0	42.8	37.2
hyperuricemia (%)	5.78	41.2	40.8	46.2
low HDL-C (%)	4.63	31.4	31.6	28.3
male gender (%)	42.7	39.2	32.8	27.3
age >60 ys (%)	23.0	20.0	24.7	27.1
no statin therapy (%)	88.3	12.9	16.4	9.0
Males				
high Lp(a) (%)	21.4	29.4	—	—
hypertension (%)	25.8	86.7	87.8	87.8
diabetes (%)	5.94	20.1	20.4	14.7
smoking (%)	11.9	64.5	69.4	78.7
obesity (%)	9.71	46.1	51.0	39.0
hyperuricemia (%)	5.64	43.9	46.9	49.1
low HDL-C (%)	4.58	28.8	30.6	25.4
male gender (%)	—	—	—	—
age >60 ys (%)	23.2	17.2	25.1	27.3
no statin therapy (%)	86.6	11.7	18.4	9.20
Females				
high Lp(a) (%)	24.1	37.9	—	—
hypertension (%)	23.6	86.0	86.4	86.2
diabetes (%)	4.59	15.8	14.6	12.1
smoking (%)	7.27	67.7	69.9	75.9
obesity (%)	10.6	39.4	38.8	36.2
hyperuricemia (%)	5.88	39.4	37.9	44.8
low HDL-C (%)	4.67	33.0	32.0	29.3
male gender (%)	—	—	—	—
age >60 ys (%)	22.9	20.9	24.1	26.9
no statin therapy (%)	89.3	13.6	15.5	8.66

**FIGURE 3:**
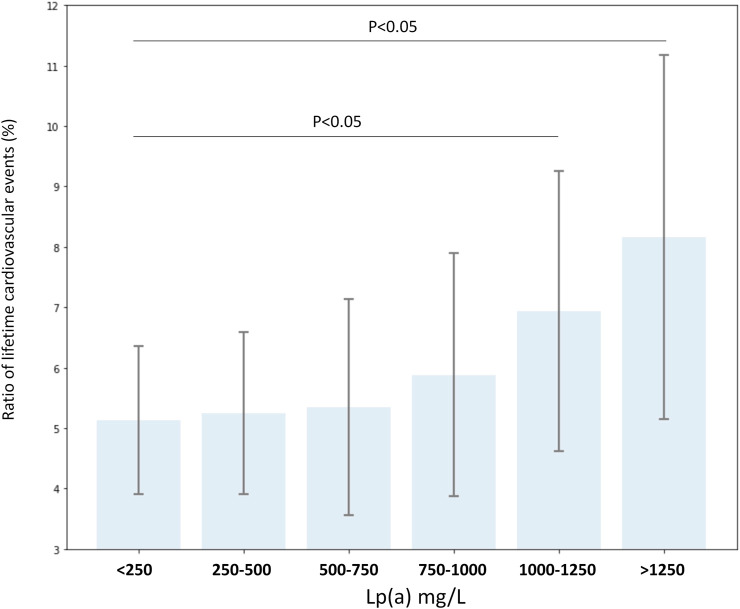
Ratio of lifetime cardiovascular events (%) according to serum lipoprotein(a) level groups in all FH and non-FH patients with known lipoprotein(a) levels.

We also calculated the impact of risk factors on hazard ratios of cardiovascular diseases. Hypertension and increased LDL-C level were found to have the biggest impact, followed by smoking, diabetes, obesity, and extremely high Lp(a) in non-FH patients. In FH patients, hypertension and smoking increased cardiovascular risk significantly (Table 4). Many patients had two or more risk factors. In patients with smoking, obesity, hypertension and diabetes, the risk of CVD was 25.74 times higher; if high LDL-C level was also associated, the risk was even higher: 27.42 (data not shown). Impact of Lp(a) levels were calculated in subgroups with various Lp(a) ranges. The risk of cardiovascular diseases was significantly increased in patients with a Lp(a) level exceeding than 1,000 mg/L (Figure 3).

**TABLE 4:** Impact of individual risk factors on cardiovascular risk (hazard ratios).

	HR (healthy^a^)	HR (healthy FH^b^)
smoking	4.9–5.2	1.3–10.6
obesity	1.9–2.2	—
hypertension	10.3–10.4	4.3–9.1
diabetes	2.3–2.7	—
high LDL-C	11.3–11.9	—
extremely high Lp(a) >1,000 mg/L	1.6–6.4	—

a Hazard rations against entirely healthy patients with no overweight, non-smoking, no statin treatment, normal LDL, and HDL levels, no hypertension, no diabetes, no high LPA (392.600 patients).

b FH patients who are not overweight, non-smoking, no high blood pressure, no diabetes, no high LPA (39 patients).

## Discussion

Despite the high cumulative LDL-C burden, not all FH patients will develop CVD to the same extend, which results in wide phenotypic heterogeneity (Neefjes et al., 2011). The simultaneous presence of multiple risk factors has been shown to increase the risk of atherosclerosis. Also, high burden of risk factor clustering might be responsible for phenotypic heterogeneity both in FH and non-FH patients. In a previous study, for index FH cases, the only factor independently associated with increased risk of CV events was the presence of corneal arcus, a known marker of long-term exposition to high levels of LDL-C. In relatives with identified genetic mutations, older age, male sex, hypertension, diabetes, previous CVD, tobacco consumption and corneal arcus were all associated with increased risk of CV events. However, multivariate analysis indicated that only diabetes and tobacco consumption remained significantly associated with the risk of CV events (Silva et al., 2016). Although relation of Lp(a) to CVD and carotid artery stenosis was reported in heterozygous FH patients almost 3 decades ago (Tatò et al., 1993), the significance of elevated Lp(a) concentrations as a risk factor is still not elucidated. Several further studies reported data on cardiovascular risk factor distribution in FH, with conflicting results.

This is the first study aiming to identify CV risk factors in Hungarian FH patients diagnosed with data mining methods. In our FH population, prevalence of hypertension was extremely high (86.3%) compared to the results of some previous studies. Dyrbus et al. also reported increased prevalence of arterial hypertension in Polish patients with definite, probable and possible FH (69.4, 70.7 and 72.6%, respectively) (Dyrbuś et al., 2019). Korneva detected a 59.2% hypertension prevalence in FH patients from Karelia (Korneva et al., 2019). However, Vlad et al. found only a 50.8% prevalence of hypertension in a Romanian FH patient population (Vlad et al., 2021). Bertolini et al. detected a 16.2% and an 23.8% prevalence of hypertension in Italian FH males and females, respectively (Bertolini et al., 2013). Mehta et al. reported a 17% hypertension prevalence in a Mexican FH cohort (Mehta et al., 2021). In another previous cohort study published by Besseling et al. hypertension was found in only 11% of FH patients (Besseling et al., 2014). We found surprisingly high prevalence of smoking (66.4%), which was similar to that of the Polish FH population mentioned above (59.2, 61.7 and 50.7% in definite, probable and possible FH, respectively) (Dyrbuś et al., 2019), but only 29.5% in the Romanian (Vlad et al., 2021), 16.8% in the Karelian (Korneva et al., 2019) and 16.7% in the Mexican FH populations (Mehta et al., 2021). In a Turkish FH registry, 12.5% of FH patients were smokers, while this number was found to be 20.2% in males according to an Italian FH registry (Bertolini et al., 2013; Kayikcioglu et al., 2018). The prevalence of diabetes in our FH cohort was comparable to the prevalence found in Polish, Romanian and Mexican FH patients (Dyrbuś et al., 2019; Mehta et al., 2021; Vlad et al., 2021), but markedly increased compared to the non-FH population. Vohnout et al. detected a lower, 10.5% prevalence of diabetes in Slovakian FH patients (Vohnout et al., 2018). It must be mentioned that a previous study reported significantly decreased diabetes prevalence in FH patients, and there was an inverse relationship between the severity of the disease-causing mutations and the diabetes prevalence (Besseling et al., 2015). Differences in socioeconomic status and genetic influences may explain these conflicting results. Additionally, the deficient knowledge of patients and their relatives on FH and its impact on health as a cardiovascular risk factor might contribute to the surprisingly high prevalence of modifiable risk factors including smoking in the studied Hungarian FH patients. Therefore, widespread information, patient education and increased awareness of this condition should be of major importance (Ceska et al., 2019).

Lp(a) levels were detected only in a few previous studies. Mehta et al. found a median Lp(a) level of 30.5 mg/dl (305 mg/L) (Mehta et al., 2021) and other previous European studies reported similar values including the study of Lingenhel et al. (27.7 mg/dl; 277 mg/L) (Lingenhel et al., 1998), Alonso et al. (23.6 mg/dl; 236 mg/L) (Alonso et al., 2014). Our results are in line with these results. Recently, lower mean Lp(a) levels were found in a Japanese cohort (20.8 mg/dl; 208 mg/L) (Naito et al., 2021) and in a previous other Japanese study (21.9 mg/dl; 219 mg/L) (Tada et al., 2018), demonstrating the importance of racial differences. Significantly increased Lp(a) levels in females were previously described in FH (Alonso et al., 2008), as well as in patients with CVD (Virani et al., 2012) and PAD (Forbang et al., 2016), and in the general population (Banerjee et al., 2011). Our data are in line with these observations. The exact cause of higher Lp(a) levels in females is not fully elucidated, but apo(a) expression has been found to be modulated by several hormones including estrogens. The chromosomal region responsible for estrogen response was identified within an apo(a) enhancer located at ∼26 kilobases from the apo(a) promoter (Boffelli et al., 1999). In the studied Hungarian population, extremely high Lp(a) levels (>1,000 mg/L) significantly increased the risk of cardiovascular events. Although the relatively low number of FH patients with available Lp(a) values impeded the evaluation of the impact of high Lp(a) concentrations on cardiovascular risk in FH patients, extremely high Lp(a) level might be also a high priority risk factor in FH. Further studies on larger FH patient populations are needed to confirm these conclusions.

In the last few years, many risk equations have been developed in order to determine CV risk associated with FH in various geographic regions. Risk equations specific to FH, such as the SAFEHEART-Risk Equation, have been validated for Spanish (Pérez de Isla et al., 2017) and French (Gallo et al., 2020) populations. The MONTREAL FH score was validated in Canada (Paquette et al., 2017). To develop a similar risk equation in Hungary, data on prevalence of these individual risk factors are essential. Our study is the first one that provides information about CV risk status of a Hungarian FH cohort.

Based on our results, the extremely large burden of vascular disease in Hungarian FH patients is mainly explained by the high prevalence of several clustered risk factors (i.e., high prevalence of smoking, obesity and hypertension), though extremely high Lp(a) levels are definitely needed to manage. Patients confronting these multiple metabolic risk factors may benefit from exploring new therapeutic frontiers achieving the goal of personalized disease management (Giglio et al., 2021). Proprotein convertase subtilisin/kexin type 9 (PCSK9) inhibitors—both monoclonal antibodies and inclisiran—lower Lp(a) by 26%, but this is insufficient for individuals with very high Lp(a) levels (Spolitu et al., 2019). New agents, such as a N-Acetylgalactosamine (GalNAc) linked antisense oligonucleotides (ASO) against Lp(a) (TQJ230, trade name pelacarsen) and a small interfering RNA (siRNA) compound aimed at reducing apo(a) synthesis (AMG 890, trade name olpasiran) reduce Lp(a) levels by 80–90% with no effect on other variables (Viney et al., 2016). Depending on results of ongoing outcome trials, these agents could be helpful for both FH and non-FH patients with elevated Lp(a) levels. Still, because of the high costs of these novel therapies, strict control of other modifiable risk factors is essential. Indeed, non-smoker patients with well controlled hypertension, diabetes and LDL-C levels, with optimal body weight and diet might have the highest benefit from Lp(a) lowering treatment. These aspects might be considered when novel agents will be prescribed. In summary, despite the technological advances, traditional diligence regarding ruling out secondary factors, encouraging a healthy diet, physical activity and weight loss, along with global CVD risk factor control remain the cornerstones of FH management and cardiovascular prevention (Berberich and Hegele, 2021).

## Limitations

Some limitations of our study must be mentioned. We were unable to assess data of family history and genetic data, moreover, we could not cover 100% of the population as not everybody goes to hospital every year. Furthermore, hospital goers tended to be older and checked more frequently. Oppositely, younger patients usually had less thorough laboratory examinations and their history been asked less frequently. These tendencies mean that identifying FH patients is biased towards the elderly. It must be highlighted that measurement of Lp(a) level is not available for each patient, mostly because of financial causes and technical issues. Furthermore, serum Lp(a) level measurements are usually indicated more frequently in patients with suspected or proved cardiovascular complications.

## Conclusion

The extremely high burden of vascular disease is mainly explained by the unhealthy lifestyle of our patients (i.e., high prevalence of smoking, unhealthy diet and physical inactivity resulting in obesity and hypertension). The lack of associations between serum Lp(a) levels and atherosclerotic vascular diseases in Hungarian FH patients may be due to the high prevalence of these risk factors masking the deleterious effect of Lp(a). Therefore, encouraging lifestyle interventions, along with global control of CVD risk factors remain the cornerstones of FH management.

## Data Availability

The raw data supporting the conclusion of this article will be made available by the authors, without undue reservation.
